# Teaching fiberoptic-assisted tracheoscopy in very low birth weight infants: A randomized controlled simulator study

**DOI:** 10.3389/fped.2022.956920

**Published:** 2022-09-08

**Authors:** Monika Wolf, Berenike Seiler, Valentina Vogelsang, Luke Sydney Hopf, Parisa Moll-Koshrawi, Eik Vettorazzi, Chinedu Ulrich Ebenebe, Dominique Singer, Philipp Deindl

**Affiliations:** ^1^Department of Neonatology and Pediatric Intensive Care Medicine, University Medical Center Hamburg-Eppendorf, University Children’s Hospital, Hamburg, Germany; ^2^Department of Pediatrics, University Medical Center Hamburg-Eppendorf, University Children’s Hospital, Hamburg, Germany; ^3^Department of Anesthesiology, University Medical Center Hamburg-Eppendorf, Hamburg, Germany; ^4^Institute of Medical Biometry and Epidemiology, University Medical Center Hamburg-Eppendorf, Hamburg, Germany

**Keywords:** medical education, Peyton’s four-step approach, premature infant, surfactant replacement therapy, bronchoscopy

## Abstract

**Objective:**

We developed a fiberoptic-assisted tracheoscopy (FAST) method to avoid direct laryngoscopy during surfactant replacement therapy and compared two training approaches on a very low birth weight (VLBW) infant simulator.

**Design:**

This prospective randomized controlled study was conducted at the Department of Neonatology and Pediatric Intensive Care Medicine of the University Medical Center Hamburg-Eppendorf, Germany.

**Participants:**

We recruited physicians, trainees, students, and nurses without prior experience in endoscopic techniques.

**Interventions:**

Participants were assigned randomly to a group that received instructions according to Peyton’s Four-Step Approach and a control group that received standard bedside teaching only.

**Main outcome measures:**

Primary endpoints were the total and the component times required to place the bronchoscope and the method success.

**Results:**

We recruited 186 participants. Compared with the control group, the Peyton group had a lower mean (±standard deviation) FAST completion time (33.2 ± 27.5 s vs. 79.5 ± 47.9 s, *p* < 0.001; *d* = 1.12) and a higher FAST success rate (95% vs. 84%, *p* = 0.036, *V* = 0.18).

**Conclusion:**

After standardized training, the vast majority of novices completed FAST successfully. Peyton’s four-step approach resulted in faster and more successful performance than standardized training.

## Introduction

There has been a recent growth in alternative surfactant administration methods for preterm infants in respiratory distress. The standard method involves laryngoscopy-guided endotracheal placement of a thin catheter, expected to be narrower than a standard endotracheal tube during spontaneous breathing under continuous positive airway pressure (1–4). Less-invasive surfactant administration (LISA) has been shown to potentially reduce ventilation duration and has been suggested to improve outcomes in very low birth weight (VLBW) infants (1, 5, 6). A recent meta-analysis concludes that administration of surfactant *via* thin catheter compared with the administration *via* an endotracheal tube is associated with reduced risk of death or bronchopulmonary dysplasia (BPD), less intubation in the first 72 h, and reduced incidence of major complications and in-hospital mortality (1). LISA appears to be promising and feasible, and has been adopted in numerous locations around the world (7–11). However, whereas there is consensus about the necessity of analgesia and sedation in premature infants undergoing laryngoscopy for intubation (12), there are no standard analgesia and sedation recommendations for LISA. While medication has been reported to improve patient comfort during LISA (13), drug-associated side effects such as hypopnea or apnea, have been reported to reduce the LISA success rate (12). Weighing the benefits of a successful LISA against infant pain exposure, many operators choose not to administer medication for laryngoscopy (10).

No studies have been published on fiberoptic-assisted surfactant administration in VLBW infants. The use of flexible bronchoscopy for difficult airways is becoming a standard of care in many pediatric intensive care units around Europe and the United States. Furthermore, in light of the availability of increasingly smaller bronchoscopes and the search for alternative methods to administer surfactant, flexible bronchoscopy will probably soon find its way into the clinical care of infants with VLBW.

We developed a fiberoptic-assisted tracheoscopy (FAST) method. During FAST, a thin, flexible bronchoscope with a working channel is placed in the proximal trachea without direct laryngoscopy. Here, we tested whether FAST can be learned and applied by novice providers within a realistic time frame. We taught the method *via* two different teaching concepts with a high-fidelity VLBW infant airway simulator. The primary endpoints were FAST success and the time required to place a bronchoscope in the proximal trachea. Participants performed the task directly after this bedside teaching instructional session. Peyton group participants went through the above additional four-step instruction (14).

## Materials and methods

### Study design

This prospective study was conducted at the Department of Neonatology and Pediatric Intensive Care Medicine of the University Medical Center Hamburg-Eppendorf, Germany, from January to August 2021, under a waiver from the local ethics committee (EK-15/02/2021). We recruited physicians, trainees, students, and nurses to participate. Informed consent was obtained from institutional staff councils and the participants. Each participant completed an intake questionnaire regarding prior intubation experience, endoscope equipment knowledge, professional specialization, and training level. We excluded three participants with previous endoscopy or bronchoscopy experience. A randomization list was used to divide the participants into a Peyton (intervention) group, taught by Peyton’s four-Step Approach (15), and a control group, given standard bedside teaching (14) (BS, MW, and VV). Peyton’s instructions included: (1) instructor *demonstration* at usual speed without commenting; (2) *deconstruction* wherein an instructor performs the task slowly and comments on each step; (3) *comprehension* wherein the trainee describes each step with instructor feedback as needed; and (4) independent trainee *performance* under instructor supervision.

Instructional sessions were conducted in small groups (≤5 participants), including standardized instruction on the flexible bronchoscope and the following procedure steps: (a) oral insertion of the bronchoscope; (b) visualization of the vocal cords; and (c) controlled passage of the vocal cords into the proximal trachea. Participants were then allowed to familiarize themselves with the bronchoscope for 120 s. The instructional sessions were scheduled for 15 min for both Peyton and the control group. The timing was started when the bronchoscope tip passed the lips and stopped when it reached the proximal trachea (t_FAST_) or 180 s had elapsed. A t_FAST_ longer than 180 s was considered a FAST failure. Two intermediate times were assessed: from procedure start to vocal cord visualization (t_vc_); and from t_vc_ to bronchoscope placement in the proximal trachea (t_TR_). The video was recorded *via* the bronchoscope. The subjects completed a second questionnaire. The primary endpoints were procedure success and the time required for FAST (t_FAST_). The secondary endpoints were possible failure causes and participants’ self-assessment. Immediately after completion, participants were asked with a questionnaire how difficult they found it to learn FAST and how confident they would feel performing it.

**FIGURE 1:**
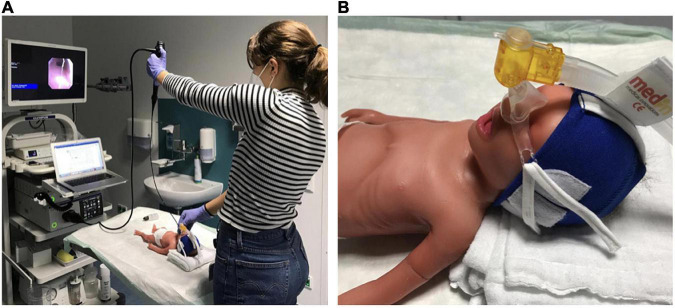
Experimental setup. **(A)** The simulator was positioned on a height-adjustable table in front of the bronchoscopy tower with the head towards the participant. Participants used a 3.1-mm-diameter Olympus EvisExera III digital video bronchoscope (BF-XP190 2711037, Tokyo, Japan) with a 1.2-mm working channel and a CV-190 video processor and xenon light source. Bronchoscope insertion was facilitated with lubricating gel. **(B)** VLBW infant simulator positioning with the head slightly elevated on a pad to optimize the view for tracheoscopy. A continuous positive airway pressure device (Fisher andPaykel, Schorndorf, Germany) was placed on the nose to simulate respiratory support during stabilization in the delivery room.

**FIGURE 2:**
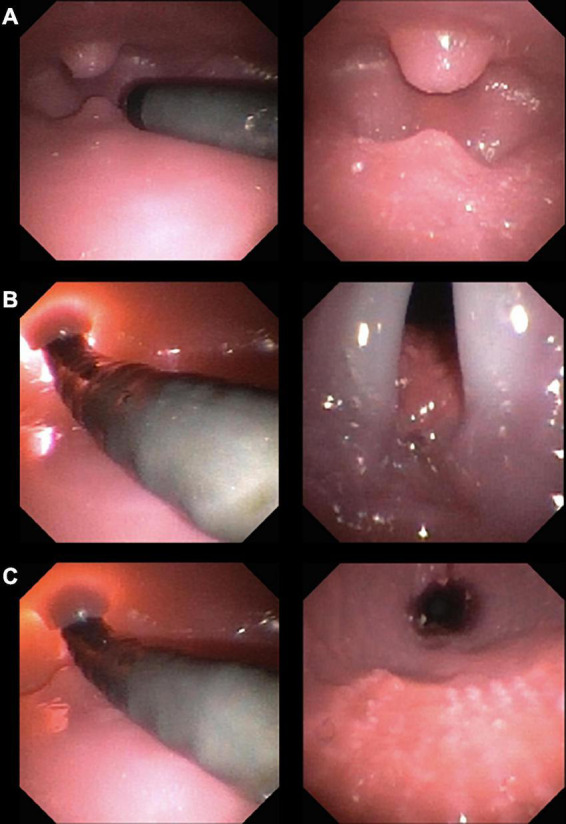
Flexible video bronchoscope route through the airway of a VLBW-infant simulator. The left side shows the lateral view of the bronchoscope, the right side the view through the bronchoscope. **(A)** The bronchoscope enters the mouth with the uvula and epiglottis visible at the “bottom” and “top”, respectively. **(B)** The bronchoscope is angulated behind the epiglottis until the larynx comes into sight. **(C)** Finally, the bronchoscope is inserted through the glottis into the trachea.

We used the VLBW infant airway simulator AirwayPaul (SimCharacters^©^, Vienna, Austria), which models an infant of 27 + 3 weeks gestational age (1,000 g) with excellent airway anatomic and functional fidelity (16). The experimental setup is shown in Figure 1. Figure 2 shows the route of the flexible video bronchoscope through the airway of the VLBW-infant simulator.

### Statistical analysis

A required minimum sample size of *N* = 89 participants per group was calculated assuming a mean t_FAST_ delta of 20 s and a standard deviation of 40 s between groups with a specified power of 0.9 and a significance level of 0.05. The random allocation sequence was generated using the sample function in R with a 50% probability (PD). Continuous and categorical variables are reported as means ± standard deviations and as category counts and percentages, respectively. Continuous variables were compared with two-sided *t*-tests, and effects were reported as Cohen’s D effect sizes. A log transformation was applied to parameters with right-skewed distributions to increase the stability of the *t*-test. Discrete data were compared between groups with the Chi-square test and effects reported as Cramer’s V effect sizes (17). We reported effect sizes to facilitate the interpretation of the importance of the results (17, 18). The *P*-values less than 0.05 were considered significant. Statistical analyses were performed in R 4.1.1 (R Core Team, Vienna, Austria).

## Results

### Participants

The characteristics of each group’s participants, including prior pediatric airway management skills, are summarized in Table 1. All the participants (*N* = 186) completed their assigned training and were analyzed for the primary outcome. The Supplementary figure 1 shows participant recruitment and analyses performed in each group.

**TABLE 1:** Participant characteristics.

Characteristic	Control *N* = 93		Peyton *N* = 93	
**Dominant hand**				
Right	85	(91.4)	87	(93.5)
Left	7	(7.5)	5	(5.4)
No specification	1	(1.1)	1	(1.1)
**Professional specialty**				
Student	66	(71.0)	58	(62.4)
Pediatrics	21	(22.6)	27	(29.0)
Adult medicine	6	(6.5)	8	(8.6)
**Professional status**				
Chief physician	−	−	1	(1.1)
Consultant	7	(7.5)	6	(6.5)
Fellow	15	(16.1)	16	(17.2)
Resident	26	(28.0)	26	(28.0)
Student	37	(39.8)	36	(38.7)
Nurse	8	(8.6)	8	(8.6)
**Assisted or watched the following procedure**				
Laryngoscopy	33	(35.5)	38	(40.9)
Intubation child	24	(25.8)	27	(29.0)
Intubation term infant	20	(21.5)	20	(21.5)
Intubation premature infant	17	(18.3)	17	(18.3)

Data are N (percentage).

### Fiberoptic-assisted tracheoscopy time

**FIGURE 3:**
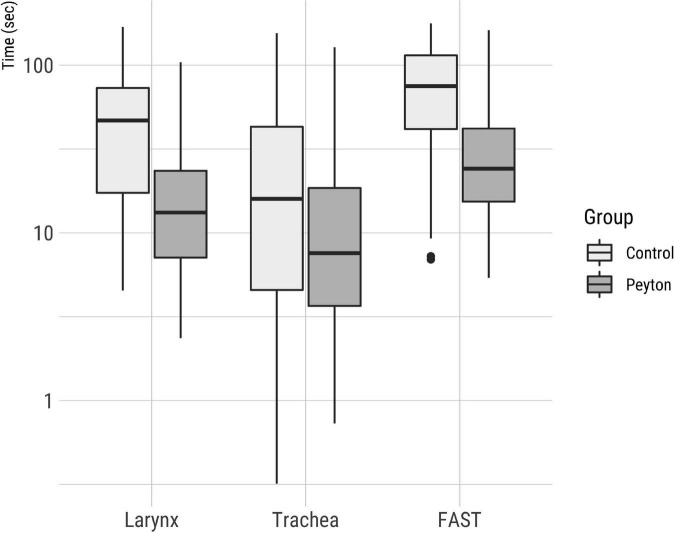
Total time for successful FAST completion on a logarithmic scale. Intermediate times for visualization of the larynx and the time elapsed between larynx visualization and endotracheal bronchoscope placement are shown.

**FIGURE 4:**
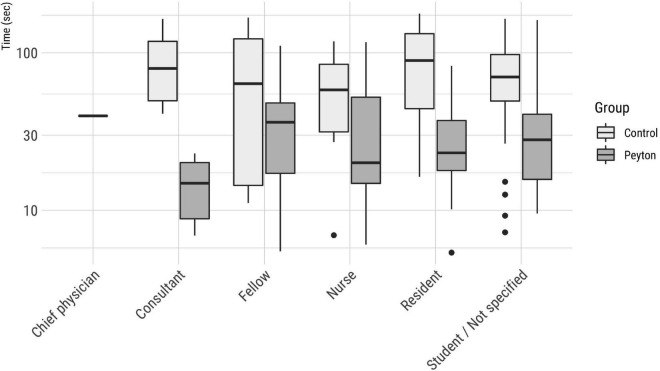
Fiberoptic-assisted tracheoscopy times according to the participants’ professional status and group.

The mean t_FAST_ with the VLBW manikin was significantly shorter in the Peyton group (33.2 ± 27.5 s) than in the control group [79.5 ± 47.9, *p* < 0.001; confidence interval (CI), 0.63–1.11; *d* = 1.12] (Figure 3). Similar differences were observed for the intermediate times t_VC_ (Peyton 19.4 ± 18.9 s vs. controls 54.1 ± 40.9 s, *p* < 0.001; CI, 0.73–1.26; *d* = 1.11) and t_TR_ (Peyton 14.7 ± 20.1 s vs. controls 29.0 ± 33.1 s, *p* = 0.002; CI, 0.01–0.90, *d* = 0.39). Figure 4 shows the t_FAST_ for the Peyton group and the controls separately according to the respective professional status of the participants. Again, participants of all subgroups performed better when instructed according to the Peyton’s approach.

### Success rate

**FIGURE 5:**
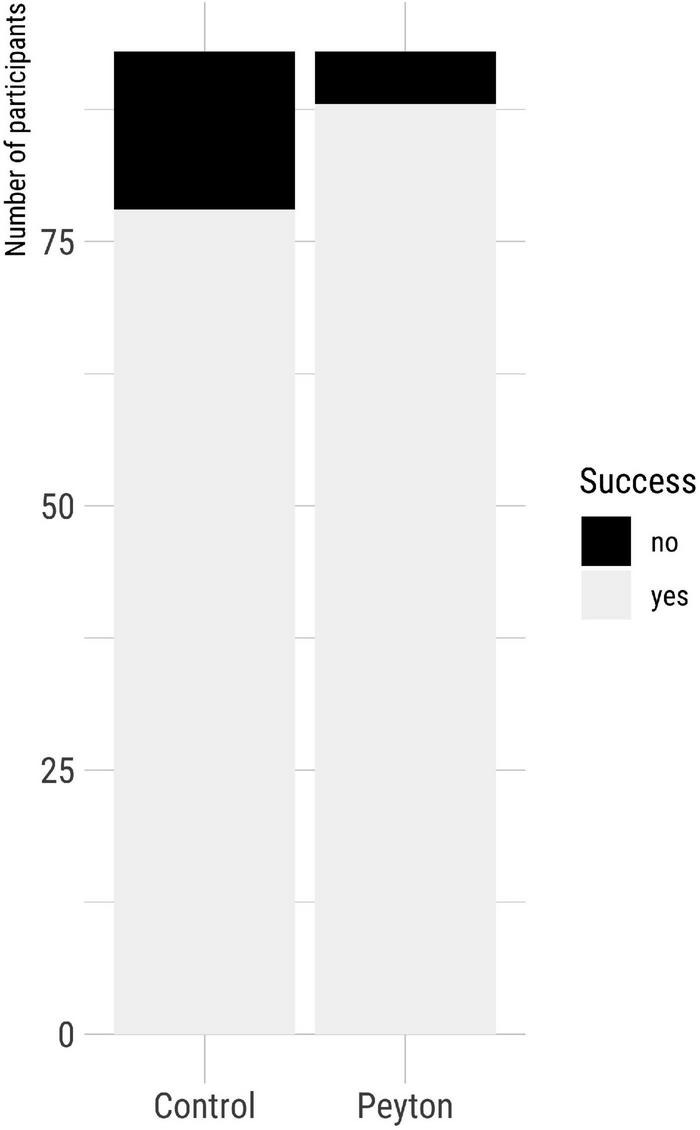
Fiberoptic-assisted tracheoscopy procedure success rate according to the group.

The FAST success rate was significantly higher in the Peyton group (95%) than in the control group (84%, *p* = 0.036, *V* = 0.18) (Figure 5). The most common causes for FAST failure in the control group were malpositioning in the esophagus, bronchoscope-handling difficulties, and lack of orientation within the airway. In the Peyton group, malpositioning in the esophagus did not occur, and other failure causes were much less frequent than in the control group (Table 2).

**TABLE 2:** FAST failure causes by group.

Cause	Control *N* = 93	Peyton *N* = 93
Malpositioning in the esophagus	12 (12.9)	0 (0)
Difficulties handling the bronchoscope	10 (10.8)	4 (4.3)
Lack of orientation	9 (9.7)	2 (2.2)
Wrong timing of kinking of the bronchoscope	7 (7.5)	4 (4.3)
Difficulty passing vocal cord level	3 (3.2)	2 (2.2)

Data are N (percentage).

### Post-participation survey

**FIGURE 6:**
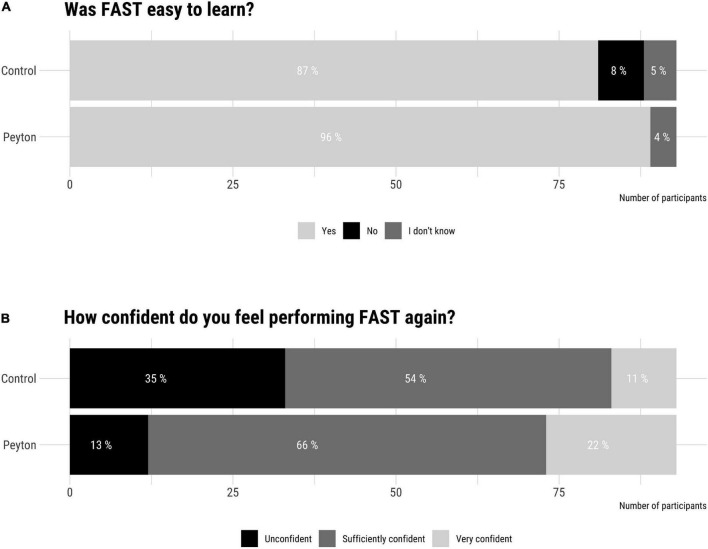
Post-participation survey results. Difficulty learning **(A)** and self-confidence to perform **(B)** Fiberoptic-assisted tracheoscopy (FAST).

Whereas 96% of Peyton group participants found FAST easy to learn, only 87% of control group did so (Figure 6). Conversely, 36% of control group participants indicated they were not confident with performing FAST again, while only 13% of Peyton group participants reported being unconfident.

## Discussion

This randomized controlled study demonstrated that FAST *via* a thin, flexible bronchoscope may be a feasible approach for LISA in a high-fidelity VLBW infant simulator. Most novice participants performed the procedure successfully after training with participants instructed with Peyton’s four-step approach showing better performance than controls.

Our finding that participants who were taught by Peyton’s approach placed the bronchoscope more quickly than participants that received only bedside teaching is in line with Krautter et al.’s results for gastric tube placement in indicating that the Peyton method is highly effective for teaching complex medical skills (14). Its main distinction from bedside teaching in the comprehension step (19) which emphasizes reflection as a crucial processing step during learning (20), provides an opportunity to correct mistakes immediately (21), and promotes long-term retention (22, 23). The graphical representation of tFAST times by professional status (Figure 4) also showed a better performance of those participants who had been instructed according to the Peyton approach, which supports the superiority of this teaching approach in the context of FAST. The time required for the Peyton approach in our study setting was similar to the time for bedside teaching, although we did not document these times precisely for each participant. However, the duration of the instructional sessions never exceeded 15 min in either case.

### Fiberoptic-assisted tracheoscopy success rate and post-participation survey

Video analysis revealed several causes for FAST failure (Table 2), including misplacement in the esophagus (12.9%) and poor orientation (9.7%), likely due to inadequate anatomical understanding of the VLBW airway (24, 25). The vast majority of participants trained using Peyton’s four-step approach found FAST easy to learn and felt confident or very confident with performing FAST again (Figure 6). We postulate that the stepwise demonstration as part of the Peyton approach may explain the participants’ better spatial and anatomical orientation when performing FAST.

### Fiberoptic-assisted tracheoscopy and surfactant replacement therapy

FAST is a modified tracheoscopy with a flexible bronchoscope that represents the first crucial step toward a clinical method for fiberoptic-assisted surfactant administration in infants with VLBW. The surfactant could be administered under visualization in infants with VLBW with respiratory distress syndrome *via* the bronchoscope’s working channel. LISA is a complex technique to learn wherein an intra-tracheal catheter is placed under direct laryngoscopy to administer surfactant during spontaneous breathing (1–3, 26, 27). Even experienced clinicians may need multiple attempts to complete it correctly (28). Bronchoscopy is commonly employed in pediatric pneumology and intensive care medicine (29, 30). Although profound (or general) analgesia is standard for pediatric bronchoscopies (30), supraglottic airway inspection with a flexible bronchoscope is feasible and safe without sedation (31, 32). We chose 180 s as the cut-off for FAST completion because preterm infants do not tolerate long and stressful procedures in the critical situation of respiratory distress. It remains unclear how taxing FAST would be for actual preterm infants. The first part of the FAST procedure, including passage of the mouth until vocal cord visualization (tVC) would probably be uncomfortable but not to be compared with the intensity and the potential pain stimulus of a laryngoscopy. During this first part of FAST (tVC), which accounted for 58% (Peyton) and 68% (Controls) of the total FAST time, relatively unimpaired spontaneous breathing might be possible. The crucial burden of FAST would be the last part with the obstruction of the trachea after the passage of the vocal cords. The relatively large diameter of the bronchoscope used for FAST (approximately the diameter of a 2.0 tube) may impair spontaneous breathing because the larynx is substantially blocked. This obstruction of the trachea by the endoscope at the moment of vocal fold passage could therefore be a relevant limitation of FAST during clinical implementation in actual preterm infants. More stable and larger preterm infants could probably tolerate FAST better than lighter and unstable patients. Surfactant administration following FAST resembles the intubation surfactant extubation (INSURE) method but does not require direct laryngoscopy and potentially obviates systemic anesthesia. Respiratory stability, hemodynamic stability, pain response, and efficacy have yet to be compared between LISA, INSURE, and FAST.

### Limitations

First, subjects and instructors were not blinded. Nevertheless, behavioral criteria were adhered to strictly during instruction. Second, participants’ laryngoscopy skills were not evaluated before inclusion into the study. As we did not re-evaluate the participants’ FAST performance, we cannot provide data regarding their long-term learning success. Thus, although the anatomical and functional fidelity of the simulator has been graded by experts as highly realistic, these results are not transferable to VLBW infants without limitations. For technical reasons, the surfactant was not administered directly into the simulator, limiting conclusiveness concerning surfactant administration. We used a VLBW airway simulator, which resembles an infant with approximately 1,000 g birth weight. Whether FAST would be possible in even lighter infants with smaller airways remains unclear. Even high-fidelity simulators cannot adequately reproduce the conditions encountered in actual patients. The major problems faced at the bedside of a living child are the child’s activity, breathing, tone, and stability. In addition, successful visualization and placement in a living infants’ larynx may require significantly more clinical training with the flexible bronchoscope. In addition, this study cannot answer whether the duration of FAST would be similar in actual patients and whether patients would clinically tolerate the manipulation with a relatively rigid bronchoscope in the mouth, pharynx, and upper airway. As the surfactant administration *via* a bronchoscope was never done in extremely preterm infants, comparisons with clinically proven administration techniques should be evaluated in future clinical studies.

## Conclusion

We conclude that FAST can be taught to novices readily on highly realistic VLBW infant airway simulators, with the Peyton method enhancing success rate and task speed relative to standard bedside training. FAST is the first crucial step toward a clinical method for fiberoptic-assisted surfactant administration in infants with VLBW with the potential advantage of reducing invasiveness compared to methods requiring direct laryngoscopy.

## Data availability statement

The raw data supporting the conclusions of this article will be made available by the authors, without undue reservation.

## Ethics statement

Ethics review and approval was not required as per local legislation and institutional requirements. The participants provided written informed consent to participate in this study.

## Author contributions

MW and PD designed the study, analyzed the data, critically discussed the results, and wrote the first draft of the manuscript. LS, PM-K, CE, and DS advised on the study design, helped discuss the results, and revised the manuscript critically for important intellectual content. BS and VV collected the data. EV advised on the statistical analysis. All authors contributed to the article and approved the submitted version.

## Supplementary material

SUPPLEMENTARY FIGURE 1 Overview of participant recruitment and analyses performed in each group.
