# Supplementary figures and images for: Teaching fiberoptic-assisted tracheoscopy in very low birth weight infants: A randomized controlled simulator study
Source: Front Pediatr. 2022 Sep 8;10:956920. doi: 10.3389/fped.2022.956920 (PMC9492998; doi:10.3389/fped.2022.956920)

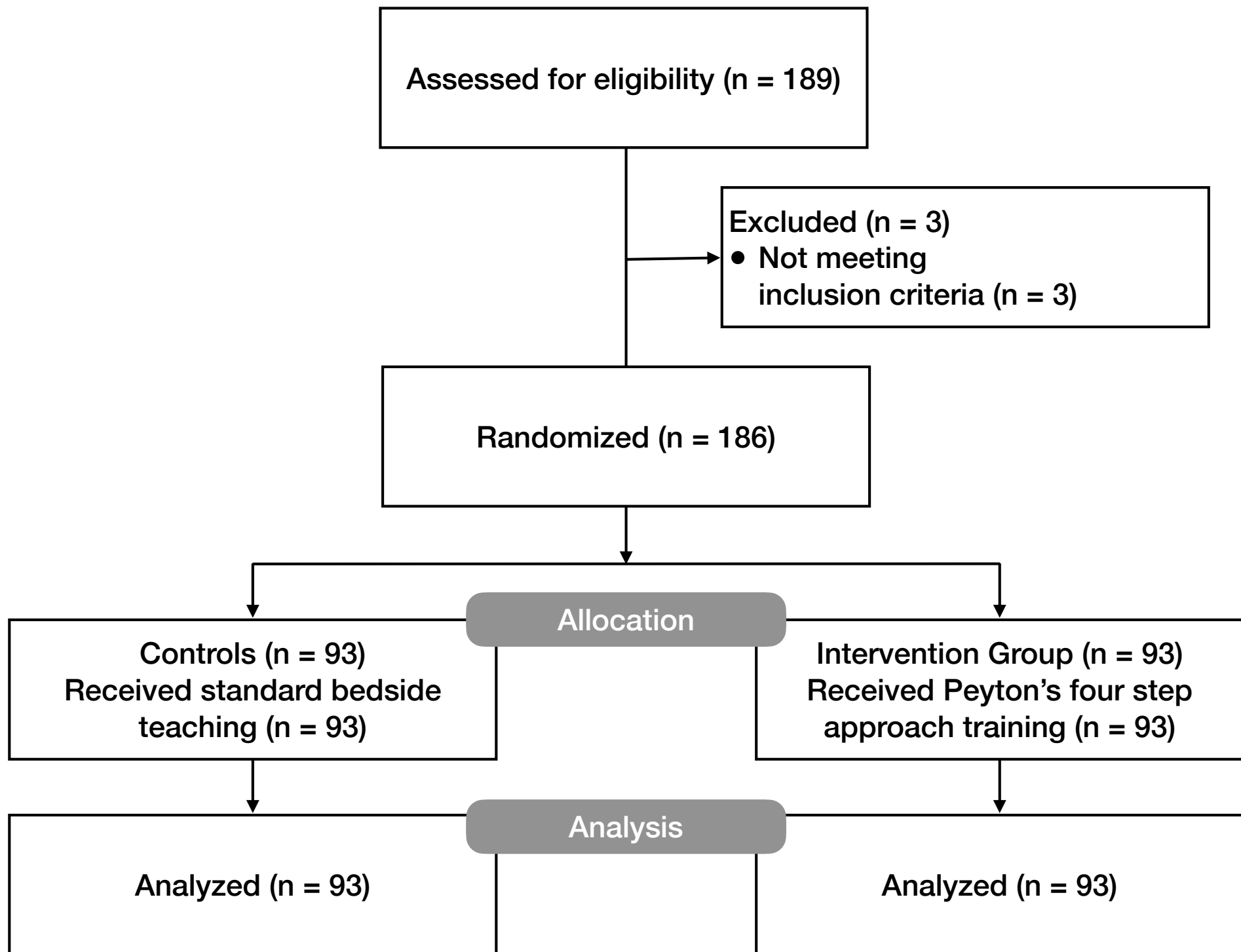

Supplement: SUPPLEMENTARY FIGURE 1 — Overview of participant recruitment and analyses performed in each group. [file Image_1.pdf]
